# Nanoscale Chemical Probing of Metal-Supported Ultrathin Ferrous Oxide via Tip-Enhanced Raman Spectroscopy and Scanning Tunneling Microscopy

**DOI:** 10.1021/cbmi.4c00015

**Published:** 2024-03-21

**Authors:** Dairong Liu, Linfei Li, Nan Jiang

**Affiliations:** † Department of Chemistry, University of Illinois Chicago, Chicago, Illinois 60607, United States; ‡ Department of Physics, University of Illinois Chicago, Chicago, Illinois 60607, United States

**Keywords:** Tip-enhanced Raman spectroscopy, Scanning tunneling microscopy, Thin-film materials, Ferrous oxide, Interfacial properties, Chemical imaging

## Abstract

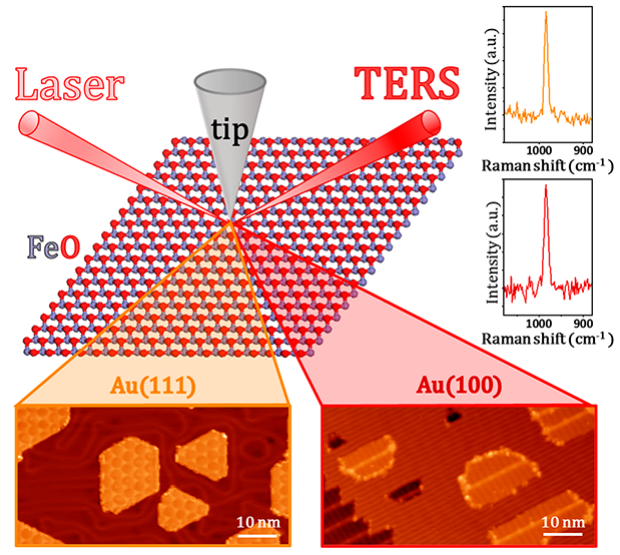

Metal-supported ultrathin ferrous oxide (FeO) has attracted immense interest in academia and industry due to its widespread applications in heterogeneous catalysis. However, chemical insight into the local structural characteristics of FeO, despite its critical importance in elucidating structure–property relationships, remains elusive. In this work, we report the nanoscale chemical probing of gold (Au)-supported ultrathin FeO via ultrahigh-vacuum tip-enhanced Raman spectroscopy (UHV-TERS) and scanning tunneling microscopy (STM). For comparative analysis, single-crystal Au(111) and Au(100) substrates are used to tune the interfacial properties of FeO. Although STM images show distinctly different moiré superstructures on FeO nanoislands on Au(111) and Au(100), TERS demonstrates the same chemical nature of FeO by comparable vibrational features. In addition, combined TERS and STM measurements identify a unique wrinkled FeO structure on Au(100), which is correlated to the reassembly of the intrinsic Au(100) surface reconstruction due to FeO deposition. Beyond revealing the morphologies of ultrathin FeO on Au substrates, our study provides a thorough understanding of the local interfacial properties and interactions of FeO on Au, which could shed light on the rational design of metal-supported FeO catalysts. Furthermore, this work demonstrates the promising utility of combined TERS and STM in chemically probing the structural properties of metal-supported ultrathin oxides on the nanoscale.

## Introduction

The importance of metal-supported ultrathin metal oxides has been well established in the past decades due to their excellent performance in heterogeneous catalysis.^1−3^ Significantly, extensive research has been carried out on ultrathin ferrous oxide (FeO) owing to its unique structural properties and high catalytic activity.^4^ Ultrathin FeO has been prepared on diverse metal surfaces, such as Pt(111),^5−12^ Pt(100),^13,14^ Pd(111),^15^ Pd(100),^16^ Mo(100),^17^ Cu(111),^18^ Cu(100),^19,20^ Cu(110),^21^ Ru(0001),^22,23^ Ag(111),^24^ Ag(100),^25−29^ and Au(111).^30−36^ These studies underscore the role of interfacial interactions with substrates in determining the morphologies and properties of ultrathin FeO films.^37−41^ In particular, local interfacial features like defects,^42^ low-coordination surface sites,^11^ and oxidation sites^43,44^ have been demonstrated to have striking impacts on the structure and reactivity of FeO. Thus, it is of significant importance to gain chemical insight into the local structural and interfacial properties of FeO on metals.

To that end, scanning tunneling microscopy (STM) provides structural characterizations with atomic precision^45,46^ but has limitations in offering comprehensive chemical information and measuring buried interfacial characteristics. While X-ray photoelectron spectroscopy (XPS) and temperature-programmed desorption (TPD) are able to trace chemical reactions,^47−50^ lack of spatial resolution hinders them from investigating site-specific structure–property relationships. In contrast, tip-enhanced Raman spectroscopy (TERS) enables chemical interrogation at the nano- and even atomic scale by coupling the spatial resolution of STM and the chemical sensitivity of Raman spectroscopy.^51−62^ TERS has proven its exceptional capacity in exploring the highly localized interfacial properties and interactions of both molecular^63−73^ and thin-film materials,^74−84^ which is of significant importance in probing the chemical activities of catalysts.^81^ In addition, TERS is sensitive to vertical interfacial interactions at the nanoscale,^85−89^ which contributes significantly to the understanding of the substrate effect of supported materials.

Here, we report the chemical probing of FeO/Au(111) and FeO/Au(100) using TERS and STM. Although the different substrate symmetries bring about distinct morphologies and moiré superstructures, we identify the same atomic structures and interfacial properties of FeO films on Au(111) and Au(100). In particular, the comparable vibrational features of FeO on both Au surfaces signify the minimal impact of the substrate symmetry on the interfacial interaction. Importantly, FeO/Au(100) exhibits a unique wrinkled structure with a weakened Raman intensity, indicating the effect of surface reconstruction on the morphology and interfacial characteristics of supported ultrathin oxides. Our research provides valuable insight into the interfacial properties and interactions of metal-supported ultrathin oxides, which could promote a deep understanding of inverse oxide-on-metal catalysts.

## Experimental Section

### 1 Synthesis of FeO/Au(111) and FeO/Au(100)

All experiments were performed in a UHV (∼1 × 10^–10^ Torr) variable-temperature system (USM1400, UNISOKU Co., Ltd.) with a home-built optical setup. Sample growth was carried out in a preparation chamber. Clean Au(111) and Au(100) surfaces were prepared by repeated cycles of Ar^+^ sputtering and annealing to ∼860 K. FeO was grown by depositing Fe onto as-prepared Au surfaces at room temperature from an Fe rod (ESPI 99.999%) using an electron-beam evaporator (ACME Technology Co., Ltd.) at an O_2_ environment (3 × 10^–7^ Torr), followed by annealing to 800 K for 15 min.

### 2 UHV-STM and TERS Characterization

All STM characterizations and TERS measurements were performed at a liquid nitrogen temperature (78 K) in the STM chamber. The homemade electrochemically etched Ag tips were used for STM imaging and TERS measurements.^90^ All STM images were acquired in constant current mode with biases applied to the sample with respect to the grounded tip. STM images were analyzed using WSxM.^91^ A 633 nm He–Ne laser (Lasos Laser GmbH) polarized parallel to the Ag tip was used for TERS measurements with a laser power of ∼5 mW. The home-built optical setup has been depicted in previous publications.^92^

## Results and Discussion

**Figure 1:**
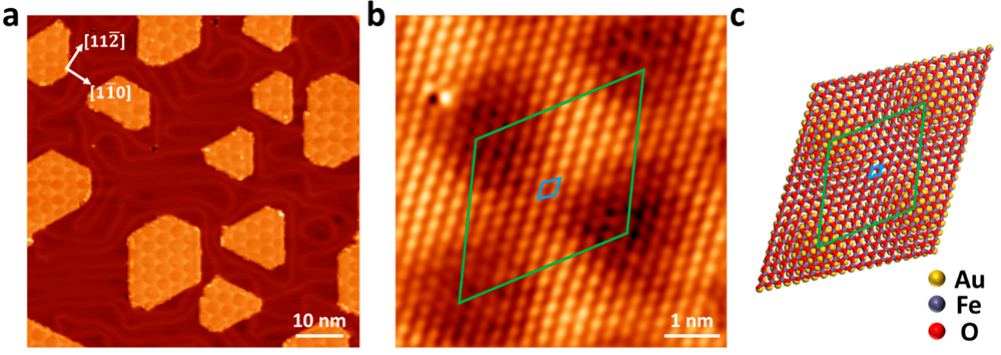
STM topography and structural model of FeO on Au(111). (a) STM topography of the FeO/Au(111) (0.4 ML). Moiré superlattice with a moiré periodicity of 3.23 ± 0.13 nm is observed on FeO nanoislands (V_bias_ = −1.5 V, I_set_ = 100 pA). (b) Atomic-resolution STM image of the FeO. A hexagonal atomic arrangement with a lattice parameter of 3.05 ± 0.13 Å is observed. The rhombuses indicate the unit cell of superlattice (green) and FeO lattice (blue), respectively (V_bias_ = −0.5 V, I_set_ = 100 pA). (c) Schematic illustration of the atomic structure of FeO/Au(111). The green and blue rhombuses indicate the unit cell of the superlattice and FeO lattice, respectively.

FeO was grown on Au(111) following the method depicted in the experimental section. As illustrated in Figure 1a, FeO nanoislands are mainly in a truncated triangle shape with a hexagonal honeycomb-like moiré superlattice. The moiré periodicity is measured to be ∼3.23 nm, consistent with our previous report.^35^ Notably, the moiré superlattice can be imaged as honeycomb or triangle patterns, indicating a bias-dependent morphology (Figure S1). The zoomed-in STM image exhibits a hexagonal atomic lattice of FeO with a lattice constant of ∼3.05 Å (blue rhombus in Figure 1b). The mismatch between the lattice of FeO and Au(111) (2.89 Å) is illustrated in Figure 1c, which exactly reproduces the observed moiré pattern as indicated with green rhombuses.^33^

**Figure 2:**
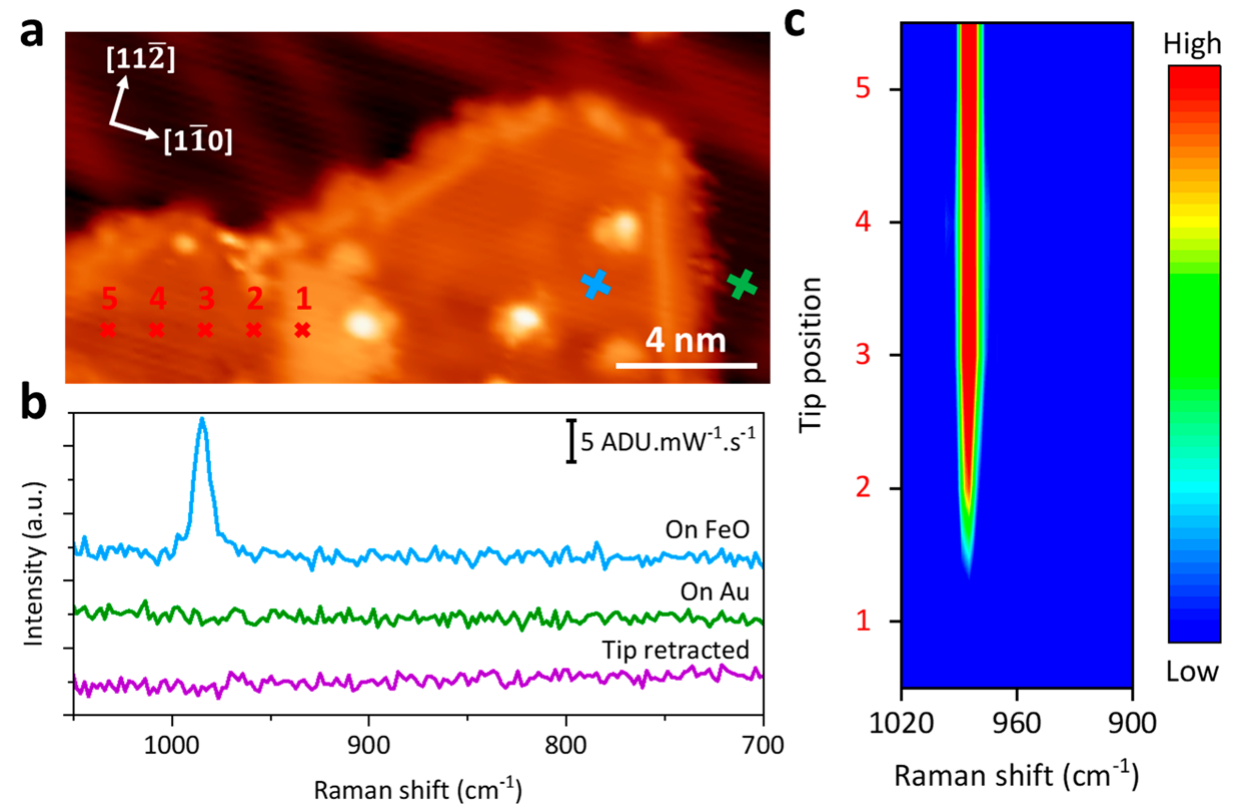
STM image and TERS characterization of FeO/Au(111). (a) STM image of FeO nanoislands on Au(111) (V_bias_ = −0.2 V, I_set_ = 100 pA). Crosses indicate the tip positions for TERS measurements shown in (b) and (c). (b) TERS spectra (−0.2 V, 2.5 nA, 10 s) acquired on the FeO (blue) and Au (green) surfaces as well as when the tip is retracted (purple). The full-range Raman spectra are presented in Figure S2c. (c) Plot of the TERS line scan (−0.2 V, 2.5 nA, 10 s acquisition time per point with a step length of 1.38 nm) along the red tip trace marked in (a).

Although STM imaging identifies the topography and atomic structure of FeO/Au(111), chemical insight into FeO nanoislands remains elusive. Herein, TERS was conducted to chemically probe FeO with nanoscale resolution.^80^ As depicted in Figure 2a and b, a featureless spectrum is observed when the tip is withdrawn from the surface (purple) or placed on the bare Au surface (green), indicating the cleanliness of the tip for TERS measurements. In contrast, a distinct 985 cm^–1^ peak is presented upon placing the tip on the FeO nanoisland (blue), suggesting the vibrational fingerprint of FeO. Figure 2c presents a waterfall plot of consecutive TERS spectra acquired along the line trace indicated by red crosses in Figure 2a. The spectra 2–5 consistently show the characteristic 985 cm^–1^ peak of FeO. In contrast, no Raman feature is presented in the first spectrum acquired on a protruding patch region on the FeO island, suggesting a nanoscale spatial resolution of our TERS measurements given a step length of 1.38 nm. This TERS observation is consistent with a particular topography of FeO on Au(111), where a strip of Au substrates sandwiched in between two FeO islands (Figure S2a) accounts for the featureless spectrum.^30^ In addition, no peak shift is observed on the FeO surface, indicating a relatively uniform chemical environment all over the FeO nanoisland.

**Figure 3:**
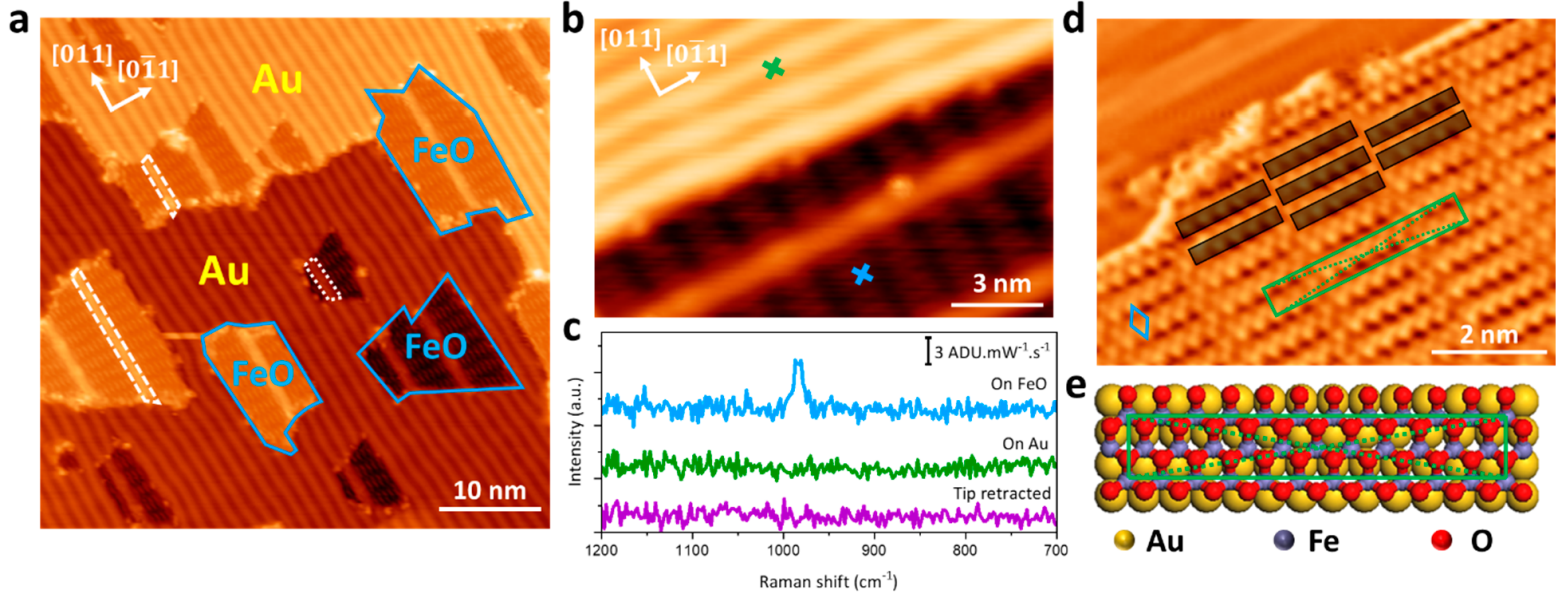
STM topography and TERS features of FeO/Au(100). (a) Large-scale image of the FeO/Au(100) (0.4 ML). The blue polygons circle out FeO nanoislands, and the white strips indicate the wrinkled structures. (b,c) TERS characterization (−0.2 V, 2.5 nA, 6 s) of FeO/Au(100) (V_bias_ = −1.2 V, I_set_ = 100 pA). (d) Derivative STM topography of the FeO/Au(100). FeO lattice (3.06 Å × 3.11 Å), moiré superlattice (3.33 nm × 0.55 nm), and brick-wall-like moiré pattern are marked by blue rhombus, green box, and black boxes, respectively (V_bias_ = −1.0 V, I_set_ = 100 pA). (e) Schematic illustration of the atomic arrangement of FeO/Au(100). The unit cell of the moiré superlattice is indicated by the green box with the dashed lines marking the center atom.

Since the substrate effect has a crucial influence on the interfacial properties of ultrathin FeO, we expanded our measurements to different Au substrates.^37^ Au(100) substrates are well-known for a reconstructed surface with a quasi-triangular lattice supported on the bulk square lattice.^93,94^ Consequently, the deposition of FeO on Au(100) could give rise to complicated and intriguing interfacial properties, providing an opportunity to deepen our understanding of the interactions between FeO and Au substrates. However, to the best of our knowledge, the growth of FeO on Au(100) remains unexplored. Following the same recipe for depositing FeO on Au(111), we observed nanoislands supported on or embedded into the terrace of Au(100) (Figure 3a) with an apparent height of ∼1 Å (Figure S3a). Notably, a moiré pattern with 2-fold symmetry was observed on nanoislands, in contrast with the hexagonal moiré pattern observed on FeO/Au(111). This distinctive moiré pattern implies the alteration of either the atomic structure of FeO or the underlying Au(100) reconstructed lattice.

To determine whether the chemical structure of FeO is changed on the top of the Au(100) substrate, TERS experiments were carried out with the same measurement condition. As shown in Figure 3b and c, a peak at 985 cm^–1^ is identified on the nanoisland (blue), excellently consistent with the TERS profile observed on FeO/Au(111). The comparable Raman features indicate that the nanoislands deposited on Au(100) share the highly similar structural properties as ultrathin FeO on Au(111). Notably, nanoislands supported on and embedded in the terrace of Au substrates show the consistent Raman features (Figures 3c and S4b), demonstrating the same chemical nature of FeO. Furthermore, no peak shift is observed in a TERS line scan taken across the nanoisland (Figure S4c and S4d), implying the relatively uniform interfacial properties of the FeO.

Following the chemical identification of FeO on Au(100), the atomic arrangement of FeO/Au(100) was resolved through high-resolution STM images. Figure 3d shows a brick-wall-like moiré pattern (highlighted by black boxes) with a rectangular moiré superlattice (green box). The moiré pattern can be assigned to a c(2 × *x*) moiré superstructure formed when a hexagonal FeO lattice is overlaid on a square surface lattice.^14,16,26^ Consequently, the 2-fold symmetric moiré superlattice suggests a transformation of Au(100) reconstruction. That is, the intrinsic quasi-triangular reconstructed lattice turns to a square Au(100) lattice upon depositing FeO on it. Moreover, the moiré superlattice of FeO/Au(100) was measured to have a periodicity of ∼3.3 nm (Figure S5), indicating a c(2 × 12) lattice that exhibits an 11-on-12 coincidence along the [011] direction of the substrate (Figure 3e; see detailed discussion in Supporting Information 6).

**Figure 4:**
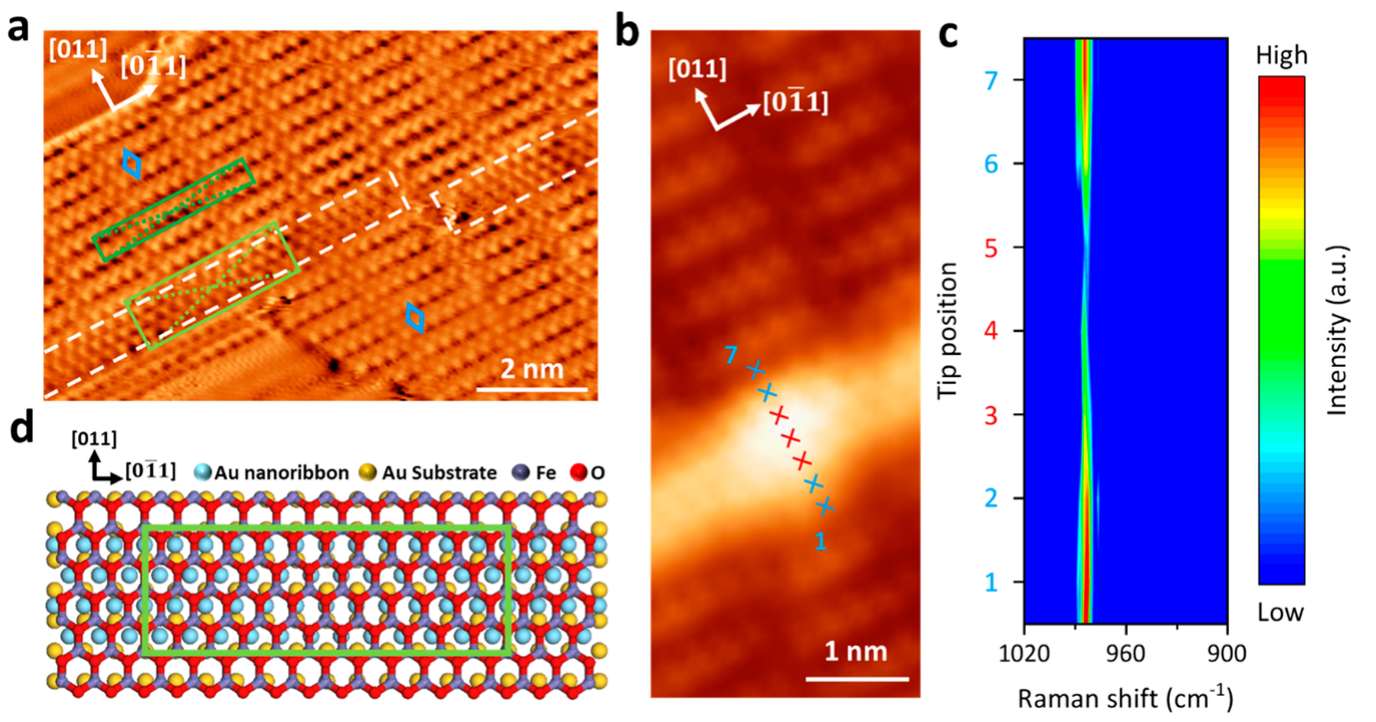
STM topography and TERS features of FeO/Au(100) wrinkled structures. (a) Atomically resolved derivative STM topography of FeO/Au(100) (V_bias_ = −1 V, I_set_ = 100 pA). The wrinkled structures are highlighted by white boxes. The moiré superlattices of FeO and FeO wrinkle are indicated by dark green and light green boxes, respectively. The identical FeO lattices at both sides of the wrinkle are marked by blue rhombuses. (b,c) Prot of TERS line scan (−0.2 V, 2.5 nA, 6 s acquisition time per point with a step length of 2.48 Å) along the tip trace in the STM image (V_bias_ = −1.2 V, I_set_ = 100 pA). (d) Schematic model of the FeO/Au(100) wrinkled structures. A moiré superlattice is highlighted by a light green box.

In addition to the predominant uniform moiré patterns, some bright strip-shaped structures are observed on FeO, as highlighted by white boxes in Figures 3a and 4a. The atomic arrangement resolved in Figure 4a depicts a c(4 × 12) moiré superlattice on the strips, indicating an 11-to-12 coincidence along the [011] direction. The same brick-wall-like superlattices are observed at both sides of the strip-shaped structures. On closer inspection, we can find a continuous hexagonal atomic arrangement across the strips (Figures 4a and S5). These topographic features rule out the possibility of line defects or domain boundaries that have been reported on FeO/Ag(100).^26^ As illustrated in Figure 4b, 4c, and S4d, TERS line scans are acquired across the strip-shape structures, which show a patent weakening of Raman intensity on the strip-shaped structures without a significant peak shift. Notably, a similar Raman feature has been reported on the wrinkles of MoS_2_ films.^77^ We thus tentatively assign the strip-shaped structure to a wrinkled FeO structure. The wrinkles are in an averaged separation of 5.25 ± 0.40 nm and a width of 1.12 ± 0.14 nm, indicating a unit cell of 4 FeO lattices supported on 20 Au lattices, where the 4 FeO lattices align with the underneath 4 Au lattices based on the moiré superlattice. Given 20% additional atoms at the top layer of Au(100) due to surface reconstruction,^94^ we tentatively propose that the formation of FeO wrinkles is correlated with the reassembly of reconstructed surface atoms of Au(100). Upon deposition of FeO, the original quasi-triangular reconstructed lattice of Au(100) turns into a square Au(100) lattice, leaving the 20% extra atoms assembling into Au nanoribbons (in 3.34 Å × 2.89 Å lattice) on top of the square Au(100) lattice with a unit cell consisting of 4-atom supported on 20-atom (Figure 4d, see details in Supporting Information 7).

## Conclusion

In conclusion, we provide a comprehensive study of the interfacial properties of ultrathin FeO on Au(111) and Au(100) substrates. Despite its distinctly different morphologies, FeO is demonstrated to have the same chemical nature on Au(111) and Au(100) by the vibrational fingerprint at 985 cm^–1^ established via nanometer-resolved TERS measurements. The consistent Raman features on FeO/Au(111) and FeO/Au(100) suggest that the surface symmetry has a minimal impact on the interfacial properties of Au-supported ultrathin FeO films. Furthermore, combined TERS and STM measurements identified a unique wrinkled structure on FeO/Au(100), highlighting the importance of local chemical characterization in probing surface variations and structural properties. Our observations provide chemical insight into the interfacial properties of Au-supported ultrathin FeO, which could contribute to investigating and devising high-efficiency metal-supported oxide catalysts.

## Supporting Information Available

Bias-dependent STM images of FeO/Au(111); STM images and full-range TERS spectra of FeO/Au(111); STM topography of FeO/Au(100) at different coverages; TERS spectra and line scan of FeO/Au(100); Line profiles of FeO/Au(100); Atomic arrangement of FeO/Au(100); The formation of Au nanoribbons and FeO wrinkles. (PDF)
