# Supplementary material for: Nanoscale Chemical Probing of Metal-Supported Ultrathin Ferrous Oxide via Tip-Enhanced Raman Spectroscopy and Scanning Tunneling Microscopy
Source: Chem Biomed Imaging. 2024 Mar 21;2(5):345–51. doi: 10.1021/cbmi.4c00015 (PMC11134605; doi:10.1021/cbmi.4c00015)
Supplement: Supplementary file 1 — im4c00015_si_001.pdf [file im4c00015_si_001.pdf]

Supporting information

Nanoscale Chemical Probing of Metal-Supported Ultrathin Ferrous Oxide via Tip-enhanced Raman Spectroscopy and Scanning Tunneling Microscopy

Dairong Liu[a], Linfei Li[a], Nan Jiang[*a,b]

[a] Department of Chemistry, University of Illinois Chicago, Chicago, IL 60607, USA

[b] Department of Physics, University of Illinois Chicago, Chicago, IL 60607, USA

*Corresponding Author

Email: njiang@uic.edu

Table of contents

1. Bias-dependent STM images of FeO/Au(111)

2. STM images and full-range TERS spectra of FeO/Au(111)

3. STM topography of FeO/Au(100) at different coverages

4. TERS spectra and line scan of FeO/Au(100)

5. Line profiles of FeO/Au(100)

6. Atomic arrangement of FeO/Au(100)

7. The formation of Au nanoribbons and FeO wrinkles

1. Bias-dependent STM images of FeO/Au(111)

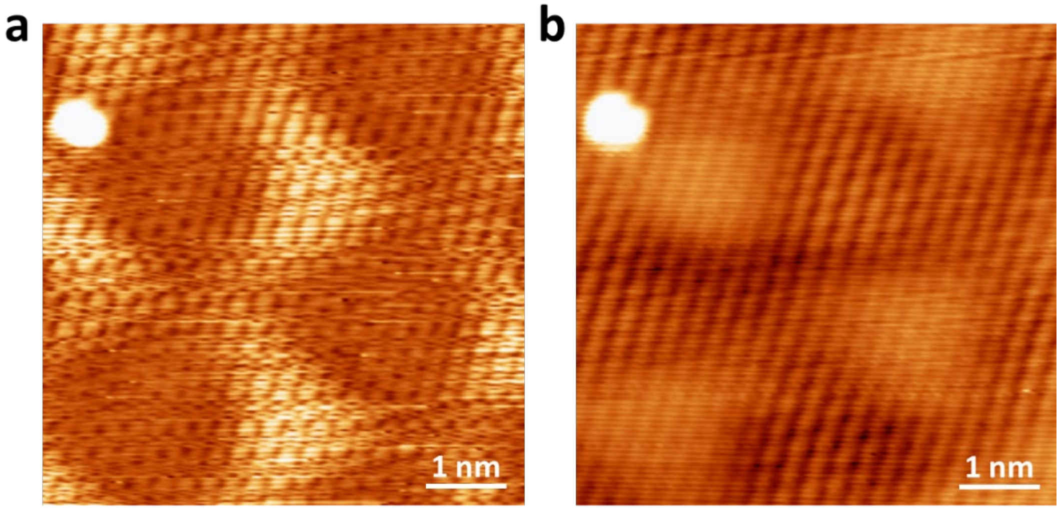

Fig. S1 Bias-dependent STM images of the same area acquired at V_{bias} = -1 V (a) and 1 V (b) and the same tunneling current I_{set} = 100 pA.

2. STM images and full-range TERS spectra of FeO/Au(111)

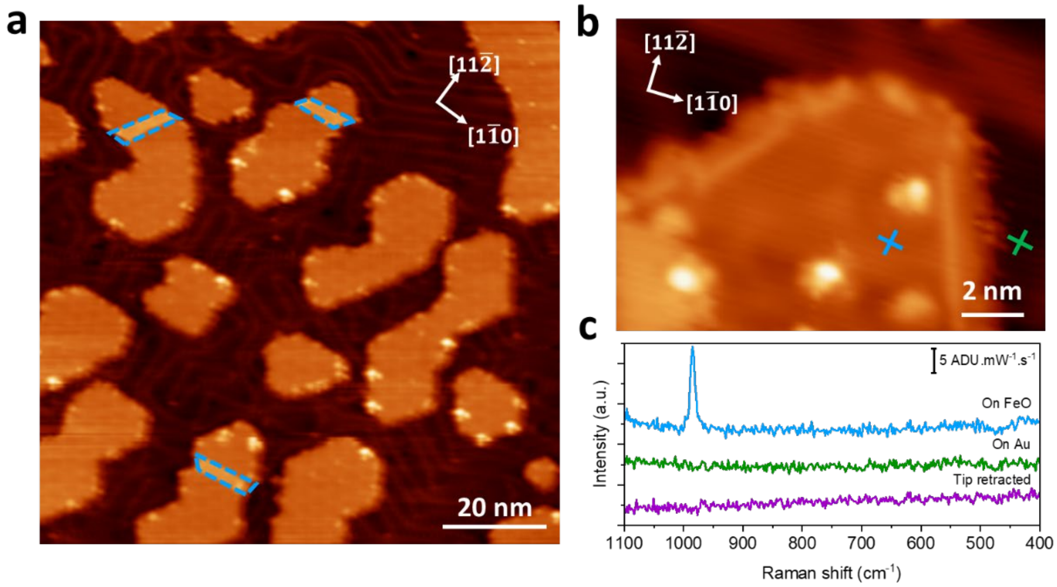

Fig. S2 STM topography and full-range TERS spectra of FeO/Au(111). (a) STM image of FeO/Au(111). The blue tetragons mark some Au islands sandwiched between FeO islands (V_{bias} = 1.2 V, I_{set} = 100 pA). (b,c) STM topography (V_{bias} = -0.2 V, I_{set} = 100 pA) and TERS spectra (-0.2 V, 2.5 nA, 10 s) acquired on the FeO (blue) and Au (green) as well as when the tip is retracted (purple).

3. STM topography of FeO/Au(100) at different coverages

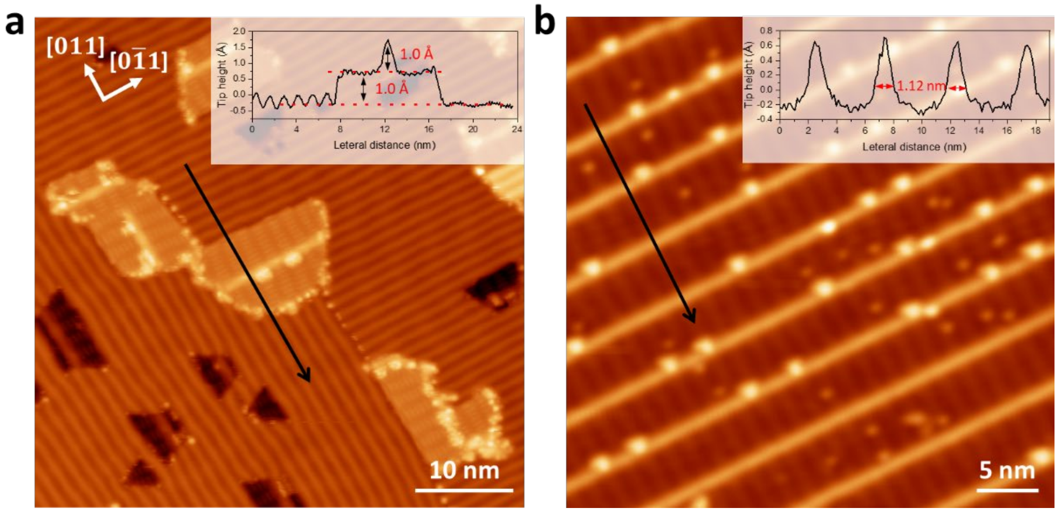

Fig. S3 STM topography of FeO/Au(100) at 0.2 ML (a) and 1 ML (b). The line profile in (a) indicates that FeO islands are around 1 Å in height and wrinkles are around 2 Å in height. The line profile in (b) indicates that the wrinkles have an average separation of 5.25 ± 0.4 nm and a width of 1.12 ± 0.14 nm. V_{bias} = -1.2 V, I_{set} = 100 pA

4. TERS spectra and line scan of FeO/Au(100)

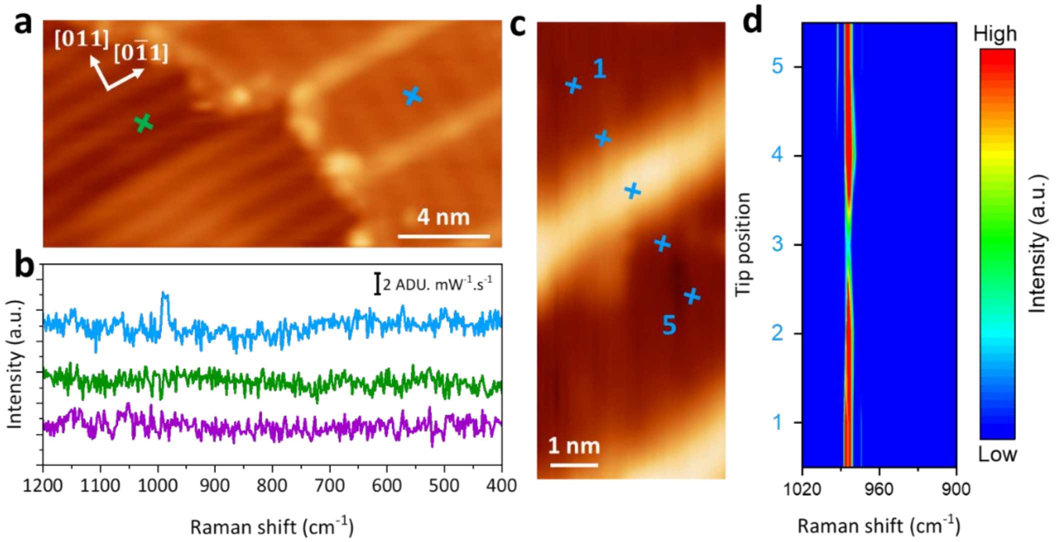

Fig. S4 TERS spectra of FeO that stand over Au terrace and TERS line scan across FeO/Au(100). (a, b) STM topography (V_{bias} = -1.2 V, I_{set} = 100 pA) and TERS spectra (-0.2 V, 2.5 nA, 6 s) acquired on the FeO (blue) and Au (green) as well as when the tip is retracted (purple). Notably, the FeO is supported on top of the Au(100) surface. (c, d) Plot of TERS line scan (-0.2 V, 2.5 nA, 6 s acquisition time per point with a step length of 1.25 nm) along the tip trace in the STM image (V_{bias} = -1.2 V, I_{set} = 100 pA).

5. Line profiles of FeO/Au(100)

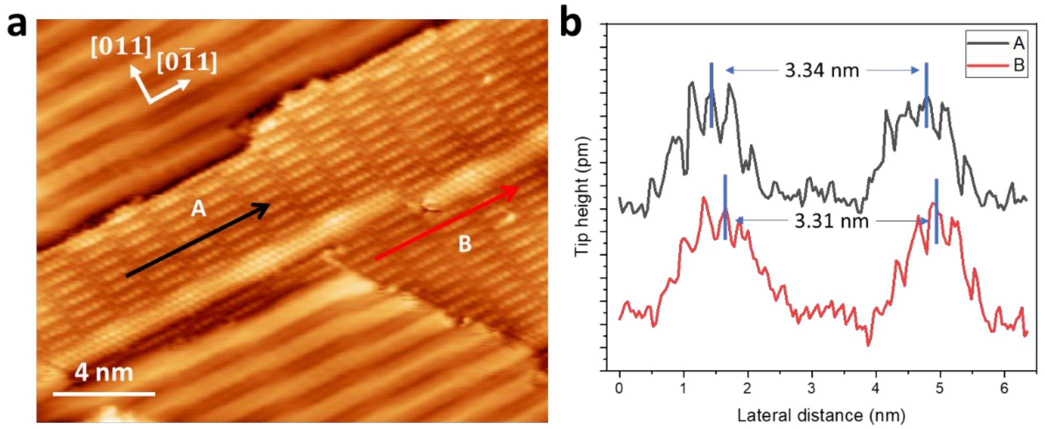

Fig. S5 STM image (a) and line profiles (b) on FeO/Au(100). The two FeO domains separated by the wrinkles have the same moiré periodicity along the $[011]_{Au}$ direction. V_{bias} = -1.2 V, I_{set} = 100 pA.

6. Atomic arrangement of FeO/Au(100)

Fig. S5 depicts a schematic model of FeO/Au(100). As resolved from STM images, FeO exhibits a quasi-hexagonal lattice with a lattice parameter of 3.06 Å along the $[0\bar{1}1]_{FeO}$ direction and 3.11 Å along the $[10\bar{1}]_{FeO}$ and $[1\bar{1}0]_{FeO}$ directions. In addition, the FeO exhibits a rectangle moiré superlattice with dimensions of 3.33 nm × 0.55 nm. Consequently, we assign this moiré superlattice as a c(2 × 12) superstructure (green box in Fig. S5), where 11 FeO(111) lattices coincide with 12 Au(100) lattices along the $[0\bar{1}1]_{FeO}$ direction. Considering the dimensions of the moiré superlattice and the lattice parameter of Au(100) surface (2.89 Å), we resolve that FeO has a lattice parameter of 3.15 Å along the $[0\bar{1}1]_{FeO}$ direction. Furthermore, 2 FeO lattices meet with 2 Au lattices along the $[\bar{2}11]_{FeO}$ directions, implying an expanded FeO lattice with a parameter of 3.34 Å along the $[10\bar{1}]_{FeO}$ and $[1\bar{1}0]_{FeO}$ directions. In short, FeO has a lattice dimension of 3.15 Å along the $[0\bar{1}1]_{FeO}$ direction and 3.34 Å along the $[10\bar{1}]_{FeO}$ and $[1\bar{1}0]_{FeO}$ directions (blue rhombus in Fig. S5), which matches with the parameters resolved from the STM images.

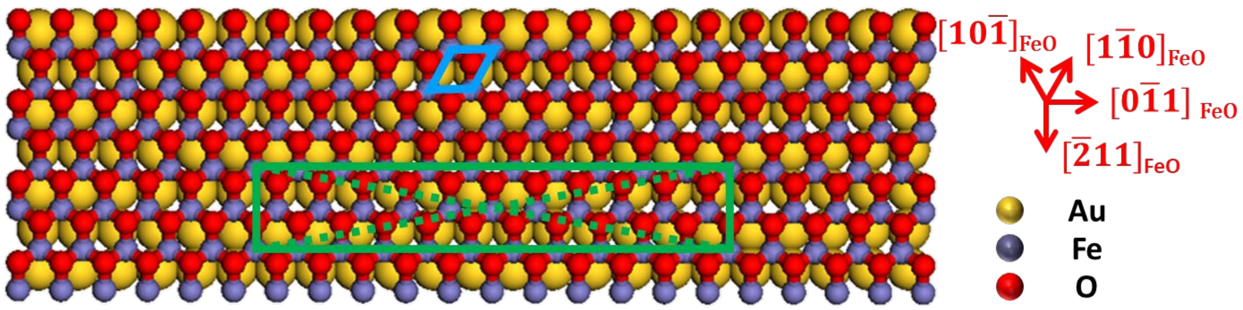

Fig. S6 Schematic illustration of FeO/Au(100). The unit cell and moiré superlattice of FeO are indicated with blue rhombus and green box, respectively. The red arrows illustrate the surface vectors of FeO.

7. The formation of Au nanoribbons and FeO wrinkles

Here, we tentatively propose a model for the formation of FeO wrinkles. As shown in Fig. S7a, the topmost layer of Au(100) crystal is intrinsically reconstructed with a quasi-triangular lattice overlaid on bulk square lattice, resulting in a (5 × N) superstructure.[1-2] As illustrated by the purple box in Fig. S7a, the ratio of Au atoms in the top layer and second layer is 6:5. That is, the top layer contains 20 % more atoms compared to the sublayer.[2] Upon deposition of FeO film, the original quasi-triangular reconstruction lattice (gold atoms in Fig. S7a) turns into a square Au(100) lattice (gold atoms in Fig. S7b). Consequently, the 20% extra atoms reassemble into an Au nanoribbon (blue atoms in Fig. S7b) on top of the square Au(100) lattice, resulting in the formation of wrinkled structures (side view of Fig. S7b). Particularly, FeO wrinkles have an average width of 1.12 ± 0.14 nm, implying they contain 4 FeO lattices in width. In addition, the FeO wrinkle exhibits a c(4 × 12) moiré superlattice, where 4 FeO lattices align with 4 underlying Au lattices (Fig. S7c). Thus, we suggest that a Au nanoribbon contains 4 reassembled Au atoms in width (side view of Fig, S7b). Moreover, the FeO wrinkle exhibits a separation of 5.25 ± 0.40 nm, which matches the separation of 20 square Au(100) lattices. Consequently, we propose that the Au surface exhibits a (20 × 1) superstructure upon FeO deposition, where 4 nanoribbon atoms are supported on 20 substrate atoms (highlighted by the purple box in Fig. S7b).

Next, we propose the atomic arrangement of the Au nanoribbon. Notably, the FeO wrinkle shares the same moiré periodicity as FeO/Au(100) along the $[0\bar{1}1]_{Au}$ direction, where 11 FeO lattices coincide with 12 underlying Au lattices (Fig. S7c). Given the lattice parameter of FeO (3.15 Å along the $[0\bar{1}1]_{Au}$ direction), we suggest that the Au nanoribbon has a lattice parameter of 2.89 Å along the $[0\bar{1}1]_{Au}$ direction. In addition, the FeO wrinkle exhibits a clear hexagonal atomic arrangement along the $[10\bar{1}]_{FeO}$ and $[1\bar{1}0]_{FeO}$ directions instead of the brick-wall-like moiré pattern exhibited on FeO/Au(100). We tentatively propose that the perfect registry of FeO and Au lattices prohibits the formation of moiré superstructure. Consequently, lattices of Au nanoribbon exhibit a 1-to-1 alignment with FeO lattices along the $[10\bar{1}]_{FeO}$ and $[1\bar{1}0]_{FeO}$ directions, implying they have a lattice parameter of 3.34 Å along those directions. In short, the Au nanoribbon has a quasi-triangular lattice in a dimension of 2.89 Å × 3.34 Å (indicated by the blue parallelogram in Fig. S7b). This mismatch between Au(100), Au nanoribbon, and FeO is shown in Fig. S7c, which aligns with the observed features in STM images.

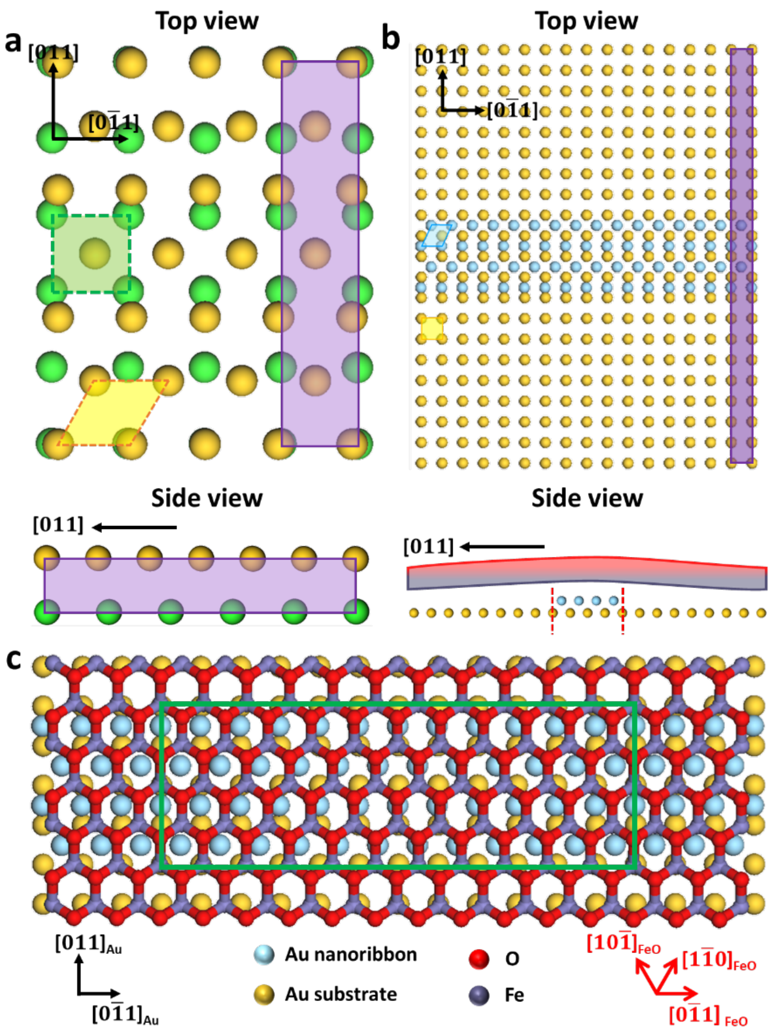

Fig. S7 Schematic models of the Au nanoribbon and FeO wrinkle structure. (a) Top and side views of the simplified schematic model of reconstruction on Au(100). The topmost layer (gold) reconstructed with a quasi-triangular lattice supported on bulk square lattice (green). The offset of the topmost layer at $[100]_{Au}$ direction is ignored. A (5 × 1) unit cell is drawn for reference in purple box. (b) Top and side views of a schematic representation of Au nanoribbon structure. The triangular lattice of Au nanoribbon (blue) supported on the square bulk Au(100) lattice (gold). For clarity, the FeO film is not shown in the top view. A (20 × 1) unit cell is indicated by a purple box, where 4 atoms are overlaid on 20 atoms. The red lines in the side view indicate the width of FeO wrinkles. (c) Schematic model for FeO wrinkled structure. The moiré superlattice of FeO wrinkle is indicated by a green box. The surface vectors of Au(100) and FeO(111) are illustrated by black and red arrows, respectively.

Reference

1. Havu, P.; Blum, V.; Havu, V.; Rinke, P.; Scheffler, M., Large-scale surface reconstruction energetics of Pt(100) and Au(100) by all-electron density functional theory. *Phys. Rev. B* **2010,** *82* (16), 161418.

2. Bengió, S.; Navarro, V.; González-Barrio, M. A.; Cortés, R.; Vobornik, I.; Michel, E. G.; Mascaraque, A., Electronic structure of reconstructed Au(100): Two-dimensional and one-dimensional surface states. *Phys. Rev. B* **2012,** *86* (4), 045426.
